# Sickness absence due to mandatory COVID-19 certificates in the workplace

**DOI:** 10.1186/s12889-023-16415-y

**Published:** 2023-08-03

**Authors:** Susanne Wanger, Enzo Weber

**Affiliations:** 1 Research Department ‘Forecasts and Macroeconomic Analyses’, Institute for Employment Research (IAB), Regensburger Straße 100, 90478 Nuremberg, Germany; 2 Chair of Empirical Economics, University of Regensburg, Regensburg, Germany

**Keywords:** Mandatory COVID-19 certificate, COVID-19, Sick leave, Workplace, Public health

## Abstract

**Background:**

As vaccines for COVID-19 became available, many countries introduced an obligation in 2021 for employees to prove their COVID-19 status at work, known in Germany as the 3G rule (vaccinated, recovered, tested). In view of the controversial debate, there was concern that employees might try to avoid providing mandatory COVID-19 certificates by taking sick leave. The aim of this study was to investigate whether mandatory COVID-19 tests in the workplace led to such an evasive response.

**Method:**

For an empirical panel analysis, we collected data from official sources and combined aggregated health insurance data on sick leave, epidemiological data on laboratory-confirmed COVID-19 infections, and vaccination rates for the German states from September 2021 to January 2022. We used a regional panel data analysis to estimate the impact of the mandatory COVID-19 certificates at the workplace on workers’ sick leave. The regional vaccination rate reflected differences in treatment intensity.

**Results:**

This study contributes to the limited evidence on the potential impact of introducing mandatory COVID-19 certificates at the workplace on sickness absence rates. In fact, our results showed that after controlling for infection rates, a one percentage point lower vaccination rate led to a 0.021 percentage point increase in the sickness absence rate when the 3G rule came into effect. This effect was measured with high statistical precision. In addition, in robustness checks, we controlled for a number of other possible influencing factors that may have affected sickness behaviours, such as time-varying labour market situations. However, the results remained robust.

**Conclusions:**

The results of our empirical panel analysis implied that mandatory COVID-19 certificates in the workplace led to evasive responses and to additional days of sick leave of a relevant magnitude. Testing obligations were meant to help contain the epidemic. However, when introducing controversial obligations, it is important to consider evasive responses and to design the rules appropriately and communicate them convincingly.

**Supplementary Information:**

The online version contains supplementary material available at 10.1186/s12889-023-16415-y.

## Introduction

The COVID-19 pandemic not only caused recessions due to declining demand but also large-scale decreases in the number of hours worked [1], particularly as a result of the widespread use of short-time working arrangements [2]. However, on the employees’ side there was also a decrease in the number of hours worked due to COVID-19. Reasons for this included COVID-19 infections [3, 4], quarantine periods [5] and the absence of parents due to the closure of schools and childcare facilities [6–8]. In Germany, just under 40 percent of establishments reported such employee-associated reductions in the number of hours worked in January 2022 [9].

In this study we examined a further possible cause of the decline in hours worked. In 2021 many countries introduced an obligation for employees to prove their COVID-19 status at work [10] to reduce the risk of infection at work, especially in cases where working from home was not possible [11].

The European Union established the Green Digital Certificate in 2021, which documents the vaccination, recovery of a person from COVID-19 or a negative COVID-19 test result carried out in the previous 48 h [12]. Certification has been introduced in many areas, e.g. for international travel or in a domestic context for access to facilities, and in some countries for access to the workplace [13]. In Germany in November 2021, the so-called 3G rule for workers to be vaccinated (**G**eimpft), recovered (**G**enesen), or tested (**G**etestet) was introduced at the workplace, i.e., employees required proof of vaccination, proof of recovery or a negative antigen (or PCR) test to gain access at work. In this way, the chain of infection could be broken at an early stage, preventing a major outbreak in an organisation.

The employee’s test result had to be available digitally or in writing. Testing in the workplace was possible under supervision. A rapid test was valid for 24 h; a PCR test for 48 h. The 3G rule and the associated controls meant that unvaccinated employees now had to undergo a COVID-19 test far more frequently on every working day. Employers had to offer their employees at least two rapid tests per week and bear the costs for them unless they worked exclusively from home. Consequently, unvaccinated workers had to pay part of the cost of testing.

As of December 2021, approximately 83% of employees were vaccinated or have recovered (2G), meaning that approximately one in six still needed regular testing. In total, approximately a quarter of the employees were tested daily at that time, as some employees with 2G status also had themselves tested [14]. This could have led to evasive reactions from employees who, for example, opposed the testing obligation or were annoyed about it. There were thus concerns that employees might try to circumvent this obligation by taking sick leave. This must also be seen in the light of the fact that debates surrounding COVID-19 policies have often been very intense and ideological.

The few findings available regarding the effects of mandatory certificates proving vaccination, recovery or a negative test in the workplace suggest that changes in behaviour can occur (see [10] for an overview). There is evidence, for instance, that restricting access to the workplace to people with COVID-19 status certificates can lead to a minority of the unvaccinated population deliberately exposing themselves to a COVID-19 infection to obtain a certificate [10]. In addition, the requirement to present a COVID19 status certificate at work may increase vaccine uptake [15, 16].

This paper contributes to the limited evidence on the potential impact of introducing mandatory COVID-19 certificates in the workplace. To the best of our knowledge, our study is the first to use a consistent empirical framework to estimate the effects on the sickness absence rate of workers. Specifically, we examined whether employees circumvented the mandatory COVID-19 certificate by taking sick leave. We identified such an effect by exploiting the substantial differences between the vaccination rates of Germany’s federal states. The more unvaccinated people there were in a state, the more strongly the sickness absence rate would react if an effect existed.

## Method

### Data collection

For the empirical analysis, we combine aggregated data at the level of the German federal states with health insurance data on the one hand and epidemiological data on the other. We collected aggregate COVID-19 pandemic-related data as well as data on sick leave absence rates from health insurance funds at the national and federal state levels. The aggregated data used are freely available for download on the official websites of the institutions.

The epidemiological data were obtained from the Robert Koch Institute (RKI), which is the government’s central scientific institution for safeguarding public health in Germany. The RKI translates laboratory-confirmed COVID-19 infection data reported to the local public health authorities into statistical data. Only cases that meet the reference definition are published as RKI statistics. These are COVID-19 cases for which there is laboratory diagnostic evidence by nucleic acid detection (e.g., PCR) or pathogen isolation. We utilised the COVID-19 infection rates in the 15–59 age group in relation to the corresponding population group [17]. Furthermore, the vaccination rates of people with basic immunisation in the 18–59 age group, in other words, those who had received two doses, were included in the estimate [18]. These data are also compiled by the RKI from the vaccination data reported by service providers (vaccination centres, mobile teams, doctors, etc.) and published as corresponding statistics. The summary of the 18–59 age group in terms of vaccination statistics is related to legislation.^1^

**Fig. 1:**
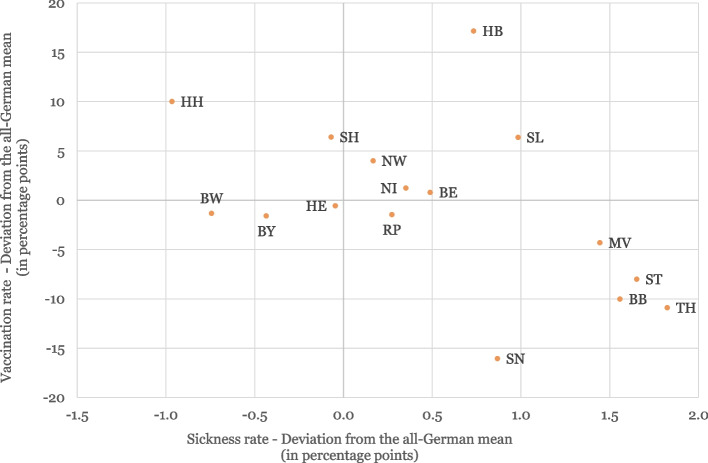
Differences in the vaccination and sick leave rates by German federal state compared to the national value (monthly averages from October 2021 to January 2022). Vaccination rates of those with basic immunisation aged 18 to 59. Abbreviations: BB, Brandenburg; BE, Berlin; BW, Baden Wurttemberg; BY, Bavaria; HB, Bremen; HH, Hamburg; HE, Hesse; MV, Mecklenburg Western Pomerania; NI, Lower Saxony; NW, North Rhine Westphalia; RP, Rhineland Palatinate; SL, Saarland; SN, Saxony; ST, Saxony-Anhalt; SH, Schleswig Holstein; TH, Thuringia

As shown in Fig. 1, regional differences were generally most pronounced between western and eastern Germany, with significantly lower vaccination rates in the eastern states (including BB, BE, MV, SN, ST, and TH). An additional file provides an overview of the different vaccination rates in the federal states at the end of September 2021 and January 2022, respectively (see Additional file 1).

**Fig. 2:**
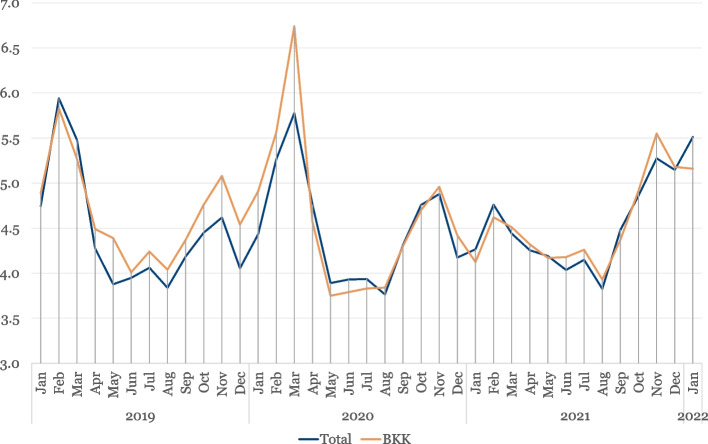
Monthly sick leave of employed health insurance members as percentages over time (January 2019 to January 2022). Deviations in the sickness absence rate may also be due to the different recording of the two statistics. The sickness absence rate on the basis of all statutory health insurance funds is calculated based on monthly average values from information on the cut-off dates on the first of the month, which are published by the Federal Ministry of Health (curve “Total”). The sickness absence rate of BKK employees corresponds to the days of incapacity to work in the entire reporting month (curve “BKK”). Abbreviation: BKK, Betriebskrankenkassen

In addition, we used data from health insurance providers on sickness absence rates in Germany. These data are based on analyses of days of sick leave of some four million employees who have statutory health insurance from a company health insurance fund (Betriebskrankenkassen, BKK) [19]. In Germany, employees are entitled to full continued payment of wages by their employer for a maximum of six weeks in the case of sickness. The sickness absence rate is calculated on the basis of days of sick leave reported and indicates the percentage of calendar days in the observation period during which each employee was unable to work on average due to sickness. Furthermore, the average number of days of sick leave is also available by major diagnostic category according to the International Statistical Classification of Diseases and Related Health Problems (ICD). The course of the sickness absence rate based on the BKK largely corresponds to that of all statutory health insurance funds in Germany (see Fig. 2); the structure of the BKK members does not differ substantially from that of the entire workforce. The figures in the additional file show an overview of the different development of sick leave rates in the federal states for the months of September 2021 to January 2022 in more detail (see Additional file 2).

The estimate was conducted using monthly data aggregated at the federal state level from September 2021 onwards. Information regarding the vaccination rates was available from this time onwards. Furthermore, from September onwards the infections were almost entirely dominated by the Delta variant. The study period ended in January 2022. In Germany, the 3G rule (vaccinated, recovered, tested) in the workplace came into force on 24 November 2021 and thus relates mainly to December and January in these data. As of 19 March 2022, the 3G rules in the workplace was relaxed again; since then, establishments have been allowed to make their own decisions about protective measures.

The effect of COVID-19 on the sickness absence rate was indeed stronger in federal states with lower vaccination rates. A 3G effect (in other words, an evasion effect) could not simply be inferred from this observation, however. It is also obvious that the sickness absence rate was higher when the vaccination rate was low because more infections would occur and because COVID-19 is more serious in unvaccinated people (although vaccination side effects could also increase the sickness absence rate).

### Data analysis

We considered this kind of correlation in a regional panel data analysis and utilised the considerable differences between the vaccination rates in the federal states. The more unvaccinated people there were in a federal state, the more strongly the sickness absence rate would respond if there was a 3G effect.

The sickness absence rate served as the dependent variable. We used federal state fixed effects to control for the general characteristics of the states (such as the sector composition of people who could potentially work from home). Time fixed effects took into account month-specific features, including seasonal effects. Furthermore, it was important to control for the regional development of the pandemic. First, the COVID-19 infection rate was used to consider differences in the infection burden. This also measured whether more infections were recorded due to the testing requirements involved in the 3G rule than had previously been the case. Second, the COVID-19 infection rate interacted with the vaccination rate, as infections can be more serious for unvaccinated people and may accordingly lead to more sick leave. Third, the base effect of the vaccination rate was also taken into account. This would also capture cases of sick leave resulting from vaccination side effects. Finally, the vaccination rate interacted with a 3G rule (December/January) dummy. This interaction measured the strength of the post-treatment effect due to the introduction of the 3G rule. The panel model is shown in Eq. (1):

1 $${sick}_{it}={c}_{1}+{c}_{2}{inf}_{it}+{c}_{3}{inf}_{it}\times {vac}_{it}+{c}_{4}{vac}_{it}+{c}_{5}{vac}_{it}\times {3G}_{t}+{\mu }_{i}+{\gamma }_{t}+{\varepsilon }_{it}$$

where c is the coefficients, $sick$ is the sickness absence rate, $inf$ is the COVID-19 infection rate, $vac$ is the vaccination rate, $3G$ is the December/January dummy, ${\mu }_{i}$ is the regional fixed effects, ${\gamma }_{t}$ is the time fixed effects and ${\varepsilon }_{it}$ is the error terms (which were verified to be serially uncorrelated). The index for the states is denoted by $i=1,\dots ,16$ and the time index by $t=1,\dots ,5$.

A 3G effect could be assumed to exist if the vaccination rate of those individuals with basic immunisation in December and January had a negative effect on the sickness absence rate exceeding that of the previous months. This procedure could be seen as a type of difference-in-differences approach with December 2021 as the treatment date. We used a special application of this approach by replacing the binary treatment with the “bite”, i.e., different vaccination rates. We borrowed this procedure from the literature that measured the effects of a nationwide minimum wage on employment; see, for instance, Card [20] or recent applications in Bauer and Weber [21] and Caliendo et al. [22]. Using regional panel data analysis, the different model estimations of the determinants of sickness absence were carried out with the statistical and econometric software package EViews version 10 (IHS Global Inc, United States). The results table shows the coefficients and t values of the different models.

## Results

The results of the estimation are shown in Table 1 (Model 1). A higher COVID-19 infection rate increased the sickness absence rate, as expected. The vaccination rate had a statistically nonsignificant negative effect, which may have been due to side effects [23]; reduction in infections due to vaccination was already controlled for by the COVID-19 infection rate variable. What was decisive for our research question was the interaction term of the vaccination rate and the 3G dummy, whose effect was estimated to be highly significant at -0.021. This means that a one percentage point lower vaccination rate led to an increase in the sickness absence rate by 0.021 percentage points when the 3G rule came into force.

**Table 1:** Panel regression results for the regional sickness absence rates and robustness checks

	Model 1		Model 2		Model 3		Model 4	
	coef	t value	coef	t value	coef	t value	coef	t value
constant	6.281	(2.83)	6.274	(2.80)	5.831	(2.22)	4.404	(1.58)
infection rate	0.269	(3.63)	0.264	(2.22)	0.217	(0.95)	0.307	(3.47)
infection rate x vaccination rate	0.0001	(0.06)	0.0001	(0.10)	0.001	(0.23)	-0.0003	(-0.31)
vaccination rate	-0.007	(-0.23)	-0.006	(-0.22)	0.001	(0.04)	-0.005	(-0.18)
vaccination rate x 3G	-0.021	(-8.67)	-0.020	(-4.29)	-0.026	(-2.34)	-0.019	(-6.78)
vaccination rate x Nov	-	-	-0.0006	(-0.11)	-	-	-	-
booster	-	-	-	-	-0.039	(-1.18)	-	-
infection rate x booster	-	-	-	-	-0.0006	(-0.16)	-	-
booster x 3G	-	-	-	-	0.043	(1.35)	-	-
unemployment rate	-	-	-	-	-	-	0.293	(2.38)
short-time rate	-	-	-	-	-	-	-0.012	(-0.22)

t values in parentheses (white period cluster)

*Abbreviations*: *coef* coefficient, *3G* 3G rule (vaccinated, recovered, tested), *Nov* November onwards dummy

To illustrate the significance of the estimation results, we considered counterfactual scenarios. If the vaccination rates of the eight states with the lowest values had equalled the average of the eight with the highest values, the sickness absence rate in December would hypothetically have been 0.07 percentage points lower due to this effect. If the vaccination rate of all the federal states had equalled the average of the top three states, the sickness absence rate would hypothetically have been 0.23 percentage points lower due to this effect. The costs associated with absenteeism are often substantially higher than the daily wage, depending on the function of the absent worker [24]. In 2020, the German Federal Institute for Occupational Safety and Health [25] estimated the costs of a day of sick leave in terms of lost production at 124 euros per employee. An extrapolation of this would result in production loss costs of between 78.3 and 257.3 million euros per day due to the higher sickness absence rate.

No indication of autocorrelation was found in the residuals. A lagged endogenous variable also played no significant role in a generalized method of moments (GMM) estimation [26].

While effects such as regional COVID-19 infection rates and vaccinations were controlled for, it is important that no unconsidered factor simultaneously altered the effect of the vaccination rate. On November 1st, 2021, two further regulations came into effect. First, unvaccinated employees would lose their entitlement to compensation for loss of earnings in accordance with the Infection Protection Act if they had to go into quarantine [27]. Second, doctors were allowed to write a sick note for respiratory illness following a telephone consultation. We therefore conducted a robustness check using an additional dummy from November onwards that interacted with the vaccination rate (Table 1, Model 2). However, this turned out to be statistically nonsignificant (the effect thus remained with the 3G dummy from December onwards). This nonsignificance of a pre-treatment indicator also provided evidence supporting the common trends assumption. Furthermore, especially from December onwards, there was a clear increase in the uptake of booster vaccines, which had a stronger impact on states whose vaccination rates were already high (double dose). As a robustness check, we additionally incorporated into the model the booster rate, the booster rate interaction term with the COVID-19 infection rate and the booster rate that interacted with the 3G dummy. The measured 3G effect then increased slightly from -0.021 to -0.026 (Table 1, Model 3). Furthermore, we controlled for the time-varying labour-market situation in the states, which may have influenced the sick leave behaviours. As proxies, we used the regional unemployment rate [28] and the regional rate of short-time workers [29]. However, the 3G effect changed only marginally (Table 1, Model 4).

We also conducted our panel analysis using days of sick leave according to major diagnostic category. The BKK data on the days of sick leave per 100 employees permitted a differentiation into eight different ICD diagnostic categories: “certain infectious and parasitic diseases (A00-B99)”, “neoplasms (C00-D48)”, “mental and behavioural disorders (F00-F99)”, “diseases of the circulatory system (I00-I99)”, “diseases of the respiratory system (J00-J99)”, “diseases of the digestive system (K00-K93)”, “diseases of the musculoskeletal system and connective tissue (M00-M99)”, and “injury, poisoning and certain other consequences of external causes (S00-T98)”. The 3G effect only appeared in the “respiratory system” category. This category may have been well suited for circumvention of the 3G rule because it includes common infectious diseases such as the cold and flu, and there was the possibility of telephone sick leave^2^ in the pandemic.

## Discussion

How does our study contribute to an understanding of the use of the 3G rule in the workplace during the COVID-19 pandemic? COVID-19 testing in the workplace has become a standard protective measure in German workplaces during periods of high incidence and widespread availability of low-cost testing. Frequent testing in the workplace can help break the chain of transmission at an early stage and protect employees from infection in the workplace. From April 2021, companies in Germany were initially required to offer their employees a COVID-19 test twice a week. However, even before that, the uptake of voluntary testing in companies was high: by the beginning of April 2021, two-thirds of employees had already received a weekly voluntary test offered by their employer [30]. In November 2021, the mandatory test offer was then replaced by the 3G rule at the workplace. The employer was now obliged to check the relevant evidence before entering the workplace, even if the 3G rule was not yet consistently adhered to by a third of the companies in the beginning [31].

There was further loss of working hours related to 3G rule, in addition to that caused by the testing itself. Our results suggest that the 3G rule led to a considerable amount of additional sick leave. Work absences due to evasive reactions to the 3G rule through increased sick leave alone were estimated at 2 million working days in Germany for the period October 2021 to February 2022. This is approximately 5% of all absences from work that were associated with increased sick leave during this period [32]. These included COVID-19 infections, COVID-19 vaccine side effects, relaxed sick leave policies and mandatory COVID-19 certificates in the workplace.

From a behavioural economics perspective, COVID-19 certificates can also be interpreted as vaccination incentives [33]. For example, the COVID-19 vaccination certificate has been shown to increase vaccination coverage [13, 15, 16]. The financial and time costs associated with testing can be a burden on employees, providing an incentive to vaccinate. This works well for some groups. However, it can have the opposite effect on those who already distrust authorities [33]. For example, 21% of the unvaccinated people said they would be even less likely to be vaccinated if the 3G rule were made mandatory in the workplace [34].

Other studies suggest that for a minority, a mandatory certificate may lead to deliberate exposure to infection [10]. In addition, mandatory COVID-19 certificates may also entail criminal behaviour as falsification and use of inaccurate vaccination or testing records increases [35].

Since the first vaccines against COVID-19 appeared, the public discourse on this issue has intensified considerably, leading to a polarisation between vaccinated and unvaccinated people [36]. In the public, it was argued on the one hand that it is ethically justifiable to require unvaccinated people to prove that they are not COVID-19 positive through reliable proof. Thus, it is justified to design individual cost–benefit incentives in such a way that the benefit of vaccination is subjectively estimated to be higher than the assumed personal costs and disadvantages [37]. On the other hand, it has been argued that a COVID-19 vaccination certificate can be seen as a form of discrimination between those who have it and can therefore exercise their constitutionally guaranteed freedoms and those who do not and whose rights have been suspended. Such a discriminatory mechanism may not be justified, as all three conditions certified by the COVID-19 certificate cannot scientifically guarantee the absence of viral infection, as infection can also occur in places to which only certified individuals have access [12].

Thus, in some regions in Germany, even the 3G rule has succeeded only to a limited extent in counteracting the reluctance to vaccinate. This is due to a number of factors, including political affiliation and regional location [38]. Particularly in federal states, where a higher proportion of the population has a low level of acceptance and trust in authorities, there is greater dissatisfaction with the 3G rule and a lower willingness to vaccinate [13, 38, 39]. These are also often federal states with very high incidences. In fact, our results also showed that the evasive reactions to sick leave when the 3G rule was introduced occurred mainly in federal states with low vaccination rates.

To minimise the potential disadvantages of a COVID-19 certificate, it should be accompanied by a combination of measures. This includes equal access to testing, vaccination and certification. Furthermore, it should be ensured that no group loses access to an everyday activity due to the requirement of certification, especially if income, health or education are affected [10]. Polarisation between vaccinated and unvaccinated individuals should be avoided so that vaccination is perceived as a health choice and not an ideological value choice [36].

## Limitations

Difference-in-difference analyses are subject to the general limitation that while common trends can be investigated pre-treatment, no definitive proof can be given for the whole sample. We checked other plausible factors that may have simultaneously altered the effect of the vaccination rate. Notwithstanding, we could not completely rule out that further relevant factors remained unconsidered.

The usual caveats apply to the generalisability and relevance of the results and conclusions of this study to other countries. Specific reasons for this include differences between countries in terms of health and epidemiological data and political as well as information strategies for COVID-19.

## Conclusion

In this paper, we quantified the impact of mandatory workplace COVID-19 certificates for employees on sickness absence by combining health insurance data and epidemiological data and analysing them at the state level for the period September 2021 to January 2022. The results of our empirical panel analysis provide evidence that workplace testing requirements lead to evasive responses. These reactions correspond to the willingness to vaccinate against COVID-19. Thus, a one percentage point lower vaccination rate, controlling for infection rates, led to a 0.021 percentage point increase in sickness-related absenteeism by the time the 3G rule came into force. These evasive reactions do not necessarily imply a violation of duties. They may have been genuine illnesses that would not otherwise have led to a sick leave note. It is therefore important to consider evasive responses when introducing controversial measures and to design the regulations appropriately and communicate them convincingly.

Future research could address mandatory certificate effects on absenteeism in other countries to ascertain the external validity of our results. One important aspect would be to increase the uptake of health certificates in regions and populations with less trust in government interventions.

### Supplementary Information

**Additional file 1: Figures A1.** Rate of full vaccination by federal states in Germany.

**Additional file 2: Figures A2.** Sickness absence rates by federal states in Germany.

## Data Availability

Already prepared, published data are used within the framework of a secondary data analysis. The data on which the results of this study are based are freely available from the Robert Koch Institute (www.rki.de), the company health insurance funds (www.bkk-dachverband.de) and the Federal Employment Agency (https://statistik.arbeitsagentur.de/). Code available upon request.

## Abbreviations

COVID-19: Coronavirus disease 2019
3G: Vaccinated, recovered, tested (*Geimpft, Genesen, Getestet*)
PCR: Polymerase chain reaction
2G: Vaccinated, recovered (*Geimpft, Genesen*)
RKI: Robert Koch Institute
BB: Brandenburg
BE: Berlin
BW: Baden Wurttemberg
BY: Bavaria
HB: Bremen
HH: Hamburg
HE: Hesse
MV: Mecklenburg Western Pomerania
NI: Lower Saxony
NW: North Rhine Westphalia
RP: Rhineland Palatinate
SL: Saarland
SN: Saxony
ST: Saxony-Anhalt
SH: Schleswig Holstein
TH: Thuringia
BKK: Company health insurance funds (*Betriebskrankenkassen*)
ICD: International Statistical Classification of Diseases and Related Health Problems
coef: Coefficient
GMM: Generalized method of moments

## Footnotes

1 The German ordinances, which regulate COVID-19 vaccinations in detail, also specify the information to be reported on the vaccinated individuals. With regard to age, information is reported in 5 age groups: 0–4 years, 5–11 years, 12–17 years, 18–59 years and 60 years and over.

2 To ease the burden on doctors’ surgeries and reduce the spread of the virus, the usual method of obtaining a sick note was relaxed temporarily during the pandemic: from November 2021 to March 2022 it was possible to get a sick note for respiratory illness without visiting the doctor in person; a doctor was able to write a sick note for up to 14 days following a telephone consultation.
