# Supplementary material for: Sickness absence due to mandatory COVID-19 certificates in the workplace
Source: BMC Public Health. 2023 Aug 3;23:1482. doi: 10.1186/s12889-023-16415-y (PMC10401870; doi:10.1186/s12889-023-16415-y)
Supplement: Supplementary file 1 — Additional file 1: Figures A1. Rate of full vaccination by federal states in Germany. [file 12889_2023_16415_MOESM1_ESM.pdf]

Additional File 1

Figures A1: Rate of full vaccination by federal states in Germany

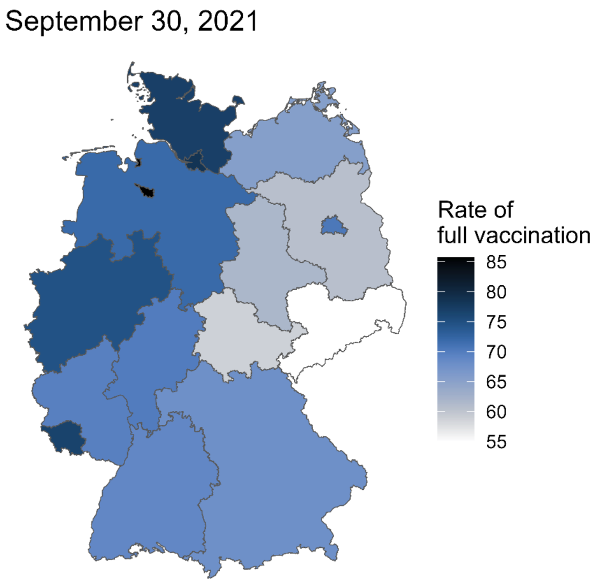

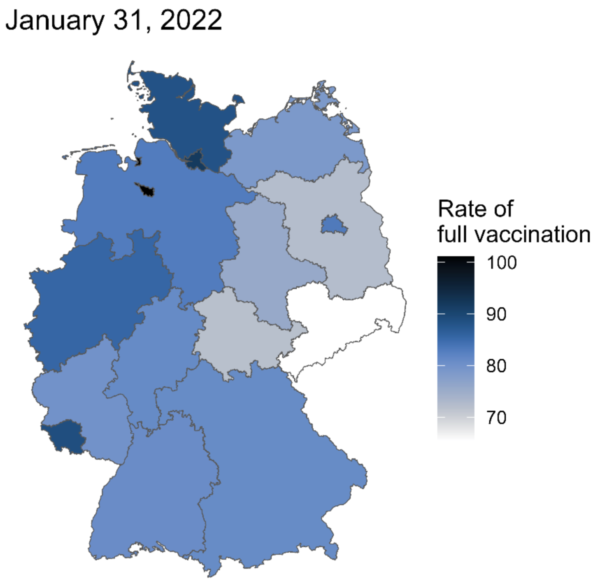

Source: RKI [18], own depiction

Note: Vaccination rates of people with basic immunisation in the 18-59 age group
