# Supplementary material for: Sickness absence due to mandatory COVID-19 certificates in the workplace
Source: BMC Public Health. 2023 Aug 3;23:1482. doi: 10.1186/s12889-023-16415-y (PMC10401870; doi:10.1186/s12889-023-16415-y)
Supplement: Supplementary file 2 — Additional file 2: Figures A2. Sickness absence rates by federal states in Germany. [file 12889_2023_16415_MOESM2_ESM.pdf]

Additional File 2

Figures A2: Sickness absence rates by federal states in Germany

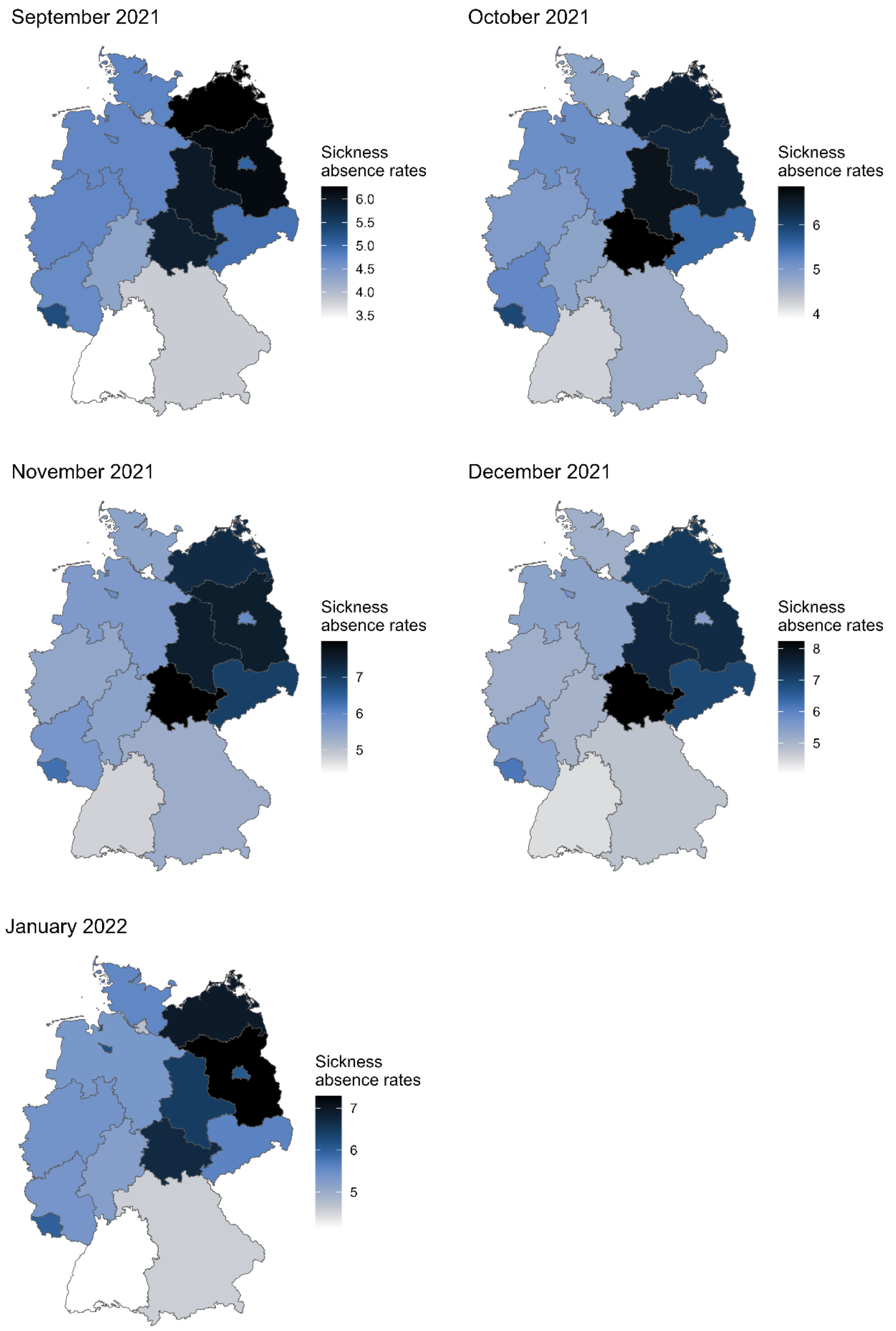

Source: BKK (Betriebskrankenkassen) [19], own depiction

Note: Sickness absence rate = Monthly sick leave of employed health insurance members as percentages over time (November 2021 to January 2022)
